# Primary Hyperaldosteronism: Epidemiology, Diagnosis, and Clinical Associations

**DOI:** 10.3390/epidemiologia7020032

**Published:** 2026-03-02

**Authors:** Christos Savvidis, Charalampos Milionis, Argyro Pachi, Athanasios Tselebis, Ioannis Ilias

**Affiliations:** 1 Department of Endocrinology, Hippokration Hospital, 11527 Athens, Greece; csendo@yahoo.gr; 2 Department of Endocrinology, Diabetes and Metabolism, Elena Venizelou Hospital, 11521 Athens, Greece; pesscharis@hotmail.com; 3 Department of Psychiatry, Sotiria Hospital, 11527 Athens, Greece; irapah67@gmail.com (A.P.); atselebis@yahoo.gr (A.T.)

**Keywords:** primary aldosteronism, secondary hypertension, aldosterone–renin ratio, adrenal venous sampling, cardiovascular morbidity, psychiatric comorbidities

## Abstract

Background/Objectives: Primary aldosteronism (PA), the leading cause of secondary hypertension, results from autonomous aldosterone hypersecretion. It is characterized by increased extracellular volume, elevated cardiac output, and greater arterial stiffness compared with essential hypertension, reflecting aldosterone-mediated hemodynamic dysregulation. The prevalence and morbidity of PA are increasingly acknowledged; however, PA continues to be underdiagnosed because of limited screening and diagnostic complexity. Methods: A narrative review was conducted using PubMed (2015–2025), with terms targeting PA epidemiology, excluding treatment-focused studies. From 971 articles, 133 relevant studies (original research studies, reviews, meta-analyses) were included, addressing prevalence, risk factors, comorbidities, genetics, and diagnostic issues. Results: PA prevalence in hypertensive populations is 5–10%, rising to 17.8% in young-onset and 20–30% in resistant hypertension. Screening indications include resistant/severe hypertension, hypokalemia, adrenal incidentaloma, young-onset disease, obstructive sleep apnea (59.8% comorbidity in hypertensive PA), and familial history, while a link may exist with papillary thyroid cancer. The aldosterone–renin ratio (ARR) is the primary screening tool, limited by assay variability and confounders (e.g., sodium intake). Confirmatory testing (such as with the saline infusion test) is often challenging to perform in routine practice. Adrenal venous sampling (AVS) is useful for subtyping unilateral (aldosterone-producing adenoma; APA; ~35–50%) vs. bilateral (idiopathic hyperaldosteronism; IHA) disease, despite technical challenges. Somatic mutations (e.g., KCNJ5, more frequent in Asians) and rare familial forms drive PA. Complications include cardiovascular events (Major Adverse Cardiovascular Events; MACE: 13.6% at 5.8 years), stroke, renal impairment (decreased eGFR, proteinuria), metabolic disorders (diabetes, obesity), and novel associations (vertebral fractures, renal stones, normal-tension glaucoma). Psychiatric comorbidities (depression/anxiety in 30–70% of patients) have been associated with central mineralocorticoid receptor effects, with sleep disturbances being prominent in females. Subclinical PA predicts hypertension and arterial stiffness. Conclusion: Improved screening protocols, standardized ARR cutoffs, and advanced imaging and genetic analyses are needed to enhance PA detection. Future research should validate cost-effective screening and clarify psychiatric-metabolic links for optimized management.

## 1. Introduction

**Figure 1:**
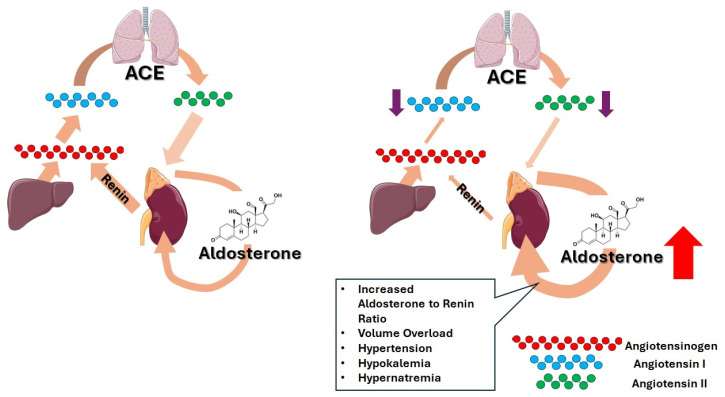
Normally (left), the renin–angiotensin–aldosterone system (RAAS) is activated when reduced renal perfusion triggers renin release, converting hepatic angiotensinogen to angiotensin I, which is then transformed by the angiotensin-converting enzyme (ACE) into angiotensin II to restore blood pressure and volume, via vasoconstriction and aldosterone secretion. In hyperaldosteronism (right), aldosterone production becomes autonomous, suppressing renin and angiotensin II activity, while causing hypertension, hypokalemia, metabolic alkalosis, and maladaptive cardiovascular/renal remodeling. Figure created with images provided by Servier Medical Art (https://smart.servier.com/, accessed on 18 November 2025), licensed under CC BY 4.0 (https://creativecommons.org/licenses/by/4.0/, accessed on 18 November 2025).

Primary aldosteronism (PA), characterized by autonomous aldosterone hypersecretion independent of the renin–angiotensin–aldosterone system (RAAS) (Figure 1), is increasingly recognized as the most common cause of secondary hypertension [1,2,3,4,5].

In the past, PA was regarded as a rare disorder, linked mainly to hypertension and hypokalemia. The prevalence of PA is substantially higher than previously realized. Conservative estimates suggest the figure is of the order of 30% [6] of all subjects with hypertension, especially when screened appropriately [6]. Improved diagnostic tools and broader screening have shown that its prevalence is higher than previously assumed, leading to its description as a “multidimensional syndrome” with a broad clinical and biochemical spectrum [4,7,8], also attributable to pathogenic somatic mutations [9]. Despite its high prevalence and its association with cardiovascular, cerebrovascular, and renal complications, PA remains largely under-recognized and under-diagnosed in clinical practice [1,2,4,7]. In this review, we provide an extensive narrative on the current understanding of the epidemiology of PA in humans. Our focus is on prevalence, risk factors, genetics, diagnostic limitations, and newer clinical associations, including those with possibly overlooked—but prevalent—psychiatric disorders.

### Search Strategy

We performed a literature search in PubMed to identify publications addressing the epidemiology of PA. The search was conducted using the following terms: “epidemiology AND hyperaldosteronism AND human NOT (treatment OR therapy)” OR “hyperaldo* AND psychiatr* AND human” OR “hyperaldosteronism AND (screening OR diagnosis) AND human NOT (treatment OR outcome OR therapy OR prognosis)”, covering the period from September 2015 to September 2025. This strategy was designed to focus on human studies reporting epidemiologic data while excluding articles primarily concerned with therapeutic interventions. The search retrieved a total of 971 articles. Titles and abstracts were screened for relevance, and full texts were reviewed when necessary. Studies were included if they provided original data, reviews, or meta-analyses pertaining to the prevalence, incidence, risk factors, demographic characteristics, or clinical correlates of PA. Reports that primarily discussed treatment, interventional outcomes, or basic science without epidemiologic relevance were excluded. After this process, 133 publications were considered relevant and were included in the present narrative review. Given the heterogeneity of study designs and outcomes, as well as the predominance of observational data, a narrative approach was considered more appropriate than a systematic or meta-analytic methodology.

## 2. Screening and Diagnosis of Primary Aldosteronism in Humans

The primary screening tool for PA, the aldosterone-to-renin ratio (ARR), is subject to various confounding factors such as sex, age, posture, and sodium intake, which can affect its accuracy [10,11]. This has led researchers to propose the establishment of population-derived, assay-specific ARR cutoffs for optimal diagnostic value [12]. The lack of standardized diagnostic protocols and interpretation challenges contributes to the dramatic under-diagnosis of PA [13]. Despite its significant association with increased cardiovascular morbidity and mortality, compared to essential hypertension, screening rates for PA remain low [4,14]. Early and accurate diagnosis and subtyping are crucial to guide appropriate management, including mineralocorticoid receptor antagonist therapy or adrenalectomy. In the Korean Endocrine Society’s 2023 guideline, the importance of early ARR screening is emphasized due to the wide prevalence variation (5.9% to 34%) and the under-detection in primary care settings [15]. In fact, hypokalemia is present in fewer than 40% of PA cases, indicating that potassium levels alone are insufficient as a screening criterion. Modalities and approaches for the screening and diagnosis of PA, according to various authorities, are presented in Table 1.

Screening for Primary Aldosteronism: The initial step in diagnosing PA involves identifying patients who require screening. Guidelines suggest testing patients with resistant or severe hypertension and those presenting with features such as hypokalemia, adrenal incidentaloma, or early-onset disease (<35–40 years), obstructive sleep apnea, first-degree relatives with PA, or a family history of early-onset hypertension or stroke at a young age [4,13,18,19,20,21]. PA is increasingly recognized as the most common cause of secondary hypertension [22]; however, hypokalemia is reported to be present in only 28% of PA patients [22]. Some experts recommend for universal screening in all subjects with hypertension, especially the elderly and those with chronic kidney disease, where the absolute risk is high despite low physician awareness [13,14]. The primary screening tool is the aldosterone-to-renin ratio (ARR), calculated from plasma aldosterone concentration (PAC) and plasma renin activity (PRA) or plasma renin concentration (PRC) [23,24,25,26]. The diagnostic performance of ARR can vary significantly due to factors such as patient population, diagnostic criteria, and the immunoassay methods used for aldosterone and renin measurements [23,24,26,27,28]. Automated chemiluminescence immunoassays (CLIAs) and liquid chromatography-tandem mass spectrometry (LC-MS/MS) are used, with LC-MS/MS offering improved precision and accuracy for steroid quantification, including aldosterone, potentially leading to more reliable ARR cut-offs [25,26,29,30,31]. Measurement of aldosterone using LC-MS/MS [9], which offers improved precision and accuracy, has shown that its values are 27–87% lower [9] compared with immunoassay-based techniques. This difference necessitates the use of assay-specific cutoff values [9] in screening and confirmatory testing. It is important to consider the intra-individual variability of aldosterone concentrations, which can influence case detection [32]. New approaches under investigation include evaluating sublingual microcirculatory dysfunction [33] and using urinary sodium/potassium ratio as a low-cost screening method in men [34].

One of the largest prospective studies (the PAPY study) demonstrated that applying systematic ARR-based screening with confirmatory testing identified PA in 11.2% of newly diagnosed patients with hypertension. Furthermore, 4.8% had surgically curable APA [35]. These results highlight how relying solely on biochemical suspicion or classic phenotypes (e.g., hypokalemia) leads to underdiagnosis.

A pragmatic approach to case detection [9] suggests that in high-risk patients with a suppressed renin, the diagnostic mindset should be that the patient has “PA until proven otherwise” [9]. Alternatively, for established hypertension, one suggested protocol is to add spironolactone 25 mg/day for 4 weeks; a blood pressure drop of <10 mmHg suggests PA is unlikely, while a drop > 12 mmHg indicates PA is probable [6].

Confirmatory Tests: Following a positive ARR, confirmatory tests are required to establish autonomous aldosterone secretion. Commonly recommended tests include the saline infusion test (SIT), fludrocortisone suppression test (FST), captopril challenge test (CCT), and oral sodium loading test (OSLT) [36,37]. However, the 2023 Korean Guidelines emphasize that confirmatory tests remain underused or inconsistently applied across centers, partly due to patient inconvenience and limited physician experience. In addition, technical difficulties and interpretation variability persist, especially with the SIT and FST [15]. Of note, in a Mayo Clinic cohort of over 260 patients with PA who underwent unilateral adrenalectomy, confirmatory testing coupled with AVS and imaging led to a 95.5% biochemical cure rate, underscoring the vital role of this diagnostic pathway in ensuring therapeutic success [38]. However, a recent systematic review highlighted that recommendations for these confirmatory tests are based on very low-quality evidence, suggesting a need for reconsideration of their routine use [36]. The accuracy of these tests can be impacted by the method of aldosterone measurement; for instance, mass spectrometric measurements during SIT may refine diagnostic cut-offs and improve reliability [30]. The CCT also presents challenges due to non-standardized interpretation and inconsistent results depending on the sampling time [39,40]. The ACTH stimulation test has been re-evaluated for its diagnostic utility in PA [41,42].

Subtyping and Lateralization: Accurate subtyping of PA into unilateral (e.g., aldosterone-producing adenoma [APA]) or bilateral (e.g., idiopathic adrenal hyperplasia [IAH]) disease is imperative for treatment planning. Adrenal Venous Sampling (AVS) is the gold standard for differentiating unilateral (APA~35–50%) from bilateral PA (~50–60%), despite technical challenges [5,22,43,44]. It is an invasive procedure requiring considerable technical expertise [45,46]. Challenges include the difficulty of cannulating the right adrenal vein (RAV), anatomical variations, and the need for accurate catheter placement [45,46,47,48,49,50]. In the PAPY study, centers with access to AVS diagnosed APA in 62.5% of PA cases, compared to only 37.5% in centers without AVS, highlighting its critical role in subtype differentiation [35].

Improving AVS Success: Techniques such as microcatheter use, CO_2_ venography, and intraprocedural CT can enhance success rates [39,44,46,51]. However, microcatheter use may increase the risk of right adrenal hemorrhage without improving sampling adequacy [39]. Guidelines from the Korean Endocrine Society emphasize using advanced imaging such as CT-guided AVS in cases of difficult right adrenal vein anatomy and suggest its incorporation into standardized protocols to improve success rates [15]. Rapid cortisol assays performed during AVS improve real-time confirmation of selective cannulation, reducing radiation exposure and procedure time [52,53,54,55]. Data from the Mayo Clinic indicate that cortisol gradients ≥ 5:1 between adrenal vein and IVC are reliable for confirming catheter placement, achieving 97.1% concordance between AVS and surgical outcomes [38].

Interpretation: Selectivity indexes based on cortisol ratios are commonly used to confirm successful cannulation, though other markers like metanephrines have also been explored [56,57,58,59]. However, the measurement of metanephrines is not routine (nor easily accessible) [52,53,54]. ACTH stimulation during AVS is debated; some studies suggest it may reduce incorrect lateralization and cannulation failures [60], but others indicate it may decrease unilateral results or shift lateralization to the dominant side [61,62]. The PAPY study explicitly avoided ACTH stimulation; there are concerns about ACTH altering diagnostic accuracy, as it may shift aldosterone gradients and affect lateralization accuracy [35]. The presence of concomitant subclinical cortisol hypersecretion can confound AVS interpretation [63,64,65]. Recent Korean guidelines and epidemiological data identify mild autonomous cortisol secretion as a significant confounder, especially in adrenal incidentalomas, which may affect AVS lateralization [15].

### 2.1. Imaging Modalities

Computed Tomography (CT) and Magnetic Resonance Imaging (MRI): Conventional CT and MRI can identify larger adenomas but are often insufficient to reliably differentiate APA from IAH or detect small, aldosterone-producing lesions [5,66]. In a 19-year-long Mayo Clinic study, adrenal imaging alone correctly lateralized disease in only 58.6% of cases, compared to 97.1% with AVS. This finding points to the limited diagnostic accuracy of CT/MRI alone [38]. Some guidelines suggest AVS might be omitted in young patients (<35 years) with pronounced PA (hypokalemia, elevated PA concentration) and a unilateral adenoma on imaging, but evidence indicates a high discordance rate between imaging and AVS results, especially in this younger population, making AVS essential for surgical candidates regardless of age [67,68,69]. Indeed, younger patients were among those most likely to have misleading imaging, with 35.1 years being the minimum age of patients in the group with inaccurate CT/MRI lateralization [38]. Spectral CT-derived extracellular volume (ECV) is a promising technique for differentiating aldosterone-producing nodules from non-functioning adrenal nodules [70]. Preliminary findings indicate that spectral CT may help quantify tissue characteristics such as fibrosis or vascularity associated with functional adenomas, offering a potential adjunctive role in pre-AVS evaluation [15].

Functional Imaging (PET/CT): PET/CT using [^11^C]metomidate or [^68^Ga]pentixafor is a non-invasive tool to identify unilateral PA by targeting aldosterone synthase (CYP11B2) or C-X-C motif chemokine receptor 4 (CXCR4), respectively [22,71,72,73,74,75]. The Korean Endocrine Society notes that [^11^C]metomidate PET/CT shows high sensitivity in lateralizing APA in selected patients and may complement AVS in centers with limited expertise [15]. While the concordance of PET/CT using [^11^C]metomidate with AVS for lateralization is modest, emerging evidence suggests it may equal or outperform AVS in predicting postoperative biochemical remission, making it a promising adjunct—but not a replacement—for AVS in selected patients [22]. However, [^11^C]metomidate PET/CT is available in few centers. A novel, highly selective PET tracer, [^18^F]AldoView, directly targeting CYP11B2, is currently under human evaluation for PA subtyping [76]. Although human trials are early-stage, this radiotracer has shown promise in preclinical studies by selectively binding to aldosterone-producing cells, with minimal uptake in cortisol-producing tissue [13]. These methods hold promise for circumventing the limitations of AVS. Functional PET imaging may especially benefit patients with inconclusive AVS, bilateral adrenal nodules, or contraindications to invasive procedures, as recognized in recent endocrine consensus reports [15].

### 2.2. Steroid Profiling

LC-MS/MS-based steroid profiling in peripheral and adrenal venous samples offers insights into PA pathophysiology and can aid in subtyping [29,77,78,79]. Biomarkers like 18-oxocortisol and 18-hydroxycortisol are being evaluated, though individual variability is significant [79]. Recent clinical guidelines emphasize that multi-steroid profiling can differentiate APA from IAH when used alongside AVS, as APA often shows a distinct pattern of elevated hybrid steroids, especially 18-oxocortisol and 18-hydroxycortisol [15]. The ongoing refinement of screening strategies, diagnostic tests with improved analytical methods, and advanced imaging techniques, alongside the indispensable role of AVS for lateralization, are collectively advancing the diagnosis and subtype classification of PA. While AVS remains central, steroid profiling is increasingly seen as a complementary tool that may reduce unnecessary AVS in selected patients with clear biochemical and imaging patterns [15].

## 3. Prevalence of Primary Aldosteronism

**Figure 2:**
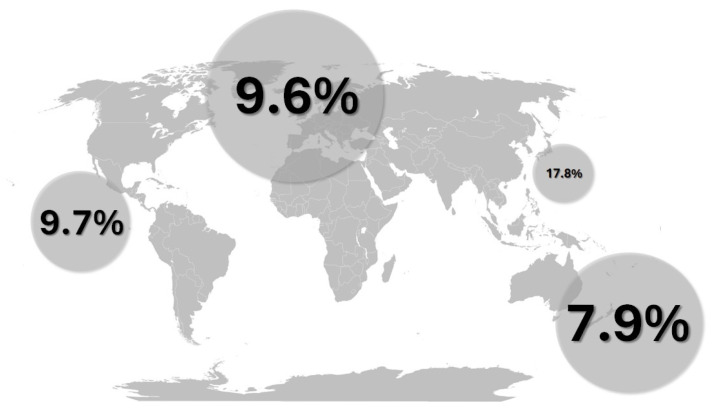
Global prevalence of primary hyperaldosteronism; drawn with data from [80]; bubble size is semi-proportional to the number of subjects from the relevant studies.

The global prevalence of PA has been estimated to be around 9.4% in hypertensive populations (95% CI: 8.3–10.5%), with a slightly higher prevalence observed in males and in South-East Asia and lower middle-income countries [80,81] (Figure 2). PA shows sexual dimorphism: women, especially of a younger age, show a higher rate of somatic KCNJ5 mutations in APA, correlating with more severe biochemical phenotypes, but improved clinical course with adrenalectomy [82,83]. By contrast, CACNA1D- and ATP1A1-gene mutations are found more often in men [82] (see below for details on genetics). The clinical presentation and identification of PA is intimately related to the biological phenomenon of aging, which occurs with a median age range of 50 years [80,84]. Although the activity of the RAAS is generally reduced with advancing age, the aldosterone to renin ratio is generally elevated in elderly patients with hypertension, due to a steeper decline in renin levels than aldosterone [11,85]. Histologically, the hallmark feature of the aging process is marked by a shift from a continuous pattern of aldosterone synthase expression in the zona glomerulosa to the emergence of aldosterone-producing cell clusters (APCCs), which feature somatic mutations to support relatively autonomous aldosterone secretion [86]. Moreover, elderly patients exhibit a reduced likelihood of successful clinical response to adrenalectomy undertaken for unilateral PA, since the long-term vascular and renal effects of chronic hypertension limit the potential for clinical cure [84,87].

However, reported prevalence rates are highly variable due to differences in study design, diagnostic criteria, and patient populations [8]. Earlier estimates suggested PA accounted for approximately 5–10% of all subjects with hypertension, but more recent studies indicate this figure may be considerably higher [7,88,89]. For example, some reports suggest a prevalence of 17.8% in young-onset hypertension [90], 8.04% in participants treated for essential hypertension (EHTN) [91], and as high as 30% in patients across different stages of hypertension when rigorous screening criteria are applied [92]. In the PAPY Study, PA was diagnosed in 11.2% of 1125 newly diagnosed hypertensive patients, with a surgically curable APA detected in 4.8% in centers using AVS, highlighting the impact of diagnostic resources on detection rates [35]. In a study rigorously adhering to the 2016 Endocrine Society guidelines, the prevalence of PA was found to be markedly high, diagnosed in 122 of 265 subjects with hypertension (46%), increasing with the number of indications for investigation [93].

Subclinical forms of PA are also highly prevalent, characterized by renin-independent aldosterone production without overt symptoms, and are negatively associated with cardiovascular health, including greater arterial stiffness, adverse cardiac remodeling, and incident hypertension, even in normotensive individuals [94,95]. Supporting this, aldosterone excess was associated with early vascular dysfunction even in normotensives with a “normal” ARR, suggesting that PA may begin as a subclinical endocrine dysregulation with long-term cardiovascular risk. Thus, recent insights emphasize that renin-independent aldosterone excess represents a continuum detectable even in normotensive individuals—reshaping PA as a progressive hormonal disorder that may precede hypertension [3].

### 3.1. Risk Factors and Screening Indications

Several clinical features are recognized as strong indicators for PA screening, as recommended by guidelines:

Resistant Hypertension: This is a major indication, with PA found in approximately 20% of patients with resistant hypertension [88,96].

Hypokalemia: Spontaneous or diuretic-induced hypokalemia is a classic sign, strongly associated with a higher prevalence of PA [97]. The incidence of PA increases continuously with decreasing potassium levels [98].

Young-Onset Hypertension: Hypertension occurring at a young age (e.g., <40 years) is a key indicator [88,99], with studies showing a high prevalence of PA (e.g., 17.8%) and a significant delay in diagnosis in this group [90].

Adrenal Incidentaloma: The presence of an adrenal incidentaloma, especially if functional, should prompt screening for PA [88,100], though non-functional lesions are more common [101,102]. The prevalence of PA in patients with adrenal incidentaloma can range from 2.4% [103] to 3.35% [104]; in patients with hypertension-associated adrenal tumors, PA prevalence was markedly higher than in those with strictly defined incidentalomas, suggesting many “non-functioning” nodules may still produce aldosterone.

Obstructive Sleep Apnea (OSA): There is a substantial co-occurrence of OSA and PA in hypertensive patients [105,106]. Meta-analyses report a 59.8% prevalence of OSA in hypertensive PA patients, with 45.4% having moderate-to-severe OSA [105]. Conversely, 11.2% of hypertensive OSA patients have PA [105]. While routine screening for PA in all OSA patients may not be cost-effective given the relatively low prevalence in OSA alone [97], targeted screening is warranted, especially in those with hypokalemia.

Severe Hypertension (Grade 2–3): The prevalence of PA increases with the grade of hypertension [91].

Familial History: A positive family history of PA is a significant risk factor, with familial aggregation and strong genetic susceptibility observed [107,108,109].

Thyroid Disorders/Nodules: PA patients show a higher prevalence of thyroid nodules compared to essential hypertension patients, suggesting the importance of screening for thyroid function and nodules in PA patients [109,110]. Also, PA is doubled in patients with atrial fibrillation compared to the general population, similar to hypo- and hyperthyroidism.

Papillary Thyroid Cancer (PTC): PTC is independently associated with a diagnosis of PA in hypertensive individuals [111], suggesting that it may be considered as a new recommendation for PA screening [112].

Despite these clear indications, the actual screening rate for PA is exceedingly low, often less than 2–3% in at-risk populations [1,3,13,113]. Barriers to wider testing include challenges in interpreting the aldosterone/renin ratio (ARR) under interfering medications and logistical issues [11]. This underdiagnosis persists even in referral centers and is further aggravated by lack of AVS access and uncertainty around ARR interpretation in patients on interfering medications [38].

### 3.2. Associated Comorbidities and Target Organ Damage

Subjects with untreated PA have an almost threefold higher risk profile of cardiovascular and cerebrovascular events and target organ damage compared to age-, sex-, and blood pressure-matched subjects with essential hypertension [1,6,114,115].

Cardiovascular Disease: PA emerges as a distinct hypertensive phenotype marked by volume expansion, aldosterone-specific cardiovascular toxicity, and characteristic hemodynamic alterations. Bioimpedance and pulse-wave analyses consistently show that, even under antihypertensive therapy, patients with PA display higher extracellular water content, greater cardiac index, elevated aortic pressures, and increased arterial stiffness compared with medicated essential hypertension [116]. These abnormalities reflect the combined effects of mineralocorticoid-driven sodium retention, nongenomic vascular inflammation and fibrosis, sympathetic activation, and altered vascular–cardiac coupling. Although systemic vascular resistance may resemble that of essential hypertension, the coexistence of excess intravascular volume and increased pulse wave velocity underscores a maladaptive hemodynamic state that may contribute to the well-documented excess cardiovascular morbidity observed in PA [117].

PA patients experience a higher incidence of major adverse cardiac events (MACEs) and adverse cardiac remodeling, including left ventricular hypertrophy (LVH) [115,118]. Long-term follow-up shows that 13.6% of PA patients developed MACEs after a median of 5.8 years [119]. Arterial stiffness, measured by brachial-ankle pulse wave velocity (baPWV), is a potential risk predictor for MACEs in PA [119]. Higher plasma aldosterone concentrations are associated with an elevated risk of aortic dissection and aneurysm, even in the absence of overt PA [120]. Emerging data also indicate that patients with suppressed renin but not meeting full PA criteria still demonstrate vascular damage, emphasizing the role of “non-classical PA” in cardiovascular pathology. Renin-independent aldosteronism is more closely associated with cardiovascular disease risk than renin-dependent aldosteronism [121].

Cerebrovascular Disease: The prevalence of stroke is considerably higher in PA patients than in those with EH [114,115]. Studies show increased risks of coronary artery disease (CAD; OR: 1.07–1.88), congestive heart failure (CHF; OR: 1.01–2.06), and stroke (OR: 1.09–1.18) in PA, highlighting the need for early and active screening [81,118]. In acute stroke patients, the pooled prevalence of PA is 5.8%, and it is more prevalent in those with atrial fibrillation (AF) or cardioembolic stroke [122,123].

Renal Impairment: Renal impairment is a frequent comorbidity in PA patients [124,125]. PA is associated with lower estimated glomerular filtration rate (eGFR) and higher prevalence of proteinuria and microalbuminuria [118,125]. Hypokalemia and increased 24 h urinary potassium excretion are key risk factors for renal damage in PA patients [125]. The uric acid to high-density lipoprotein cholesterol ratio (UHR) is inversely associated with eGFR and positively associated with chronic kidney disease (CKD) in PA patients [126]. Screening for PA is underutilized in CKD patients, potentially leading to substantial cardiovascular and renal sequelae [127].

Metabolic Disorders: PA patients have a higher risk of glycemic abnormalities (RR: 1.54) [128] and a higher prevalence of metabolic syndrome (MetS), particularly those who are overweight or obese [129]. Newly diagnosed diabetes mellitus (DM) is a risk factor for cardiocerebrovascular events in PA [130]. Obesity is an important underlying cause of PA due to the stimulatory role of the adipose tissue-derived factor, leptin [131]. Leptin stimulates the adrenal cortex to secrete aldosterone, through calcium-mediated signaling that increases CYP11B2 gene expression, which is a calcium-dependent mechanism that predominates in females [131,132]. However, the relationship between the levels of both factors is further exacerbated among premenopausal females due to the progesterone-mediated increase in mineralocorticoid receptor density in the vascular endothelium, leading to the impairment of endothelial function and salt-sensitive hypertension [133]. In addition, increased visceral and BMI values are relatively higher in IHA than APA and are mostly associated with significant metabolic risk [134]. In turn, increased BMI is an important underlying independent risk factor, among both sexes, for subjects with confirmed PA to develop depressive disorders [135] (see section on psychiatric disorders). Hypokalemic PA patients often exhibit a worse metabolic status, including higher BMI, more severe dyslipidemia, insulin resistance, and higher serum uric acid levels [136]. The prevalence of obesity and PA may be associated in circumstances leading to hypertension [134,137].

Other Associations: PA has been linked to an increased risk of vertebral fracture, especially unilateral PA (OR: 3.16) [138,139]. Patients with PA also have a higher incidence of renal stones, which tend to be larger and harder than those found in subjects with essential hypertension [140]. A novel finding suggests an 11.8% prevalence of normal-tension glaucoma (NTG) in PA patients, an elevated risk not mediated by blood pressure [141].

### 3.3. Subtypes of PA and Genetic Considerations

Differences exist between subtypes regarding comorbidities. Patients with bilateral PA (IHA) are often more obese and have higher visceral fat levels than those with unilateral PA (APA), potentially leading to higher metabolic risk despite a milder PA form [134,142]. Despite this difference in hormonal severity, studies comparing the prevalence of MetS across PA subtypes have yielded varying results. Some research has reported a higher prevalence of metabolic disorders in patients with bilateral PA compared to those with unilateral disease [61]. However, one analysis found that the two subgroups (APA and IHA) were similar regarding MetS components, although there were characteristic hormonal differences [73]. Unilateral PA, conversely, has been associated with more severe cardiac damage than bilateral PA [114]. In particular, echocardiographic evidence suggests greater left ventricular mass and wall thickness in APA, likely due to longer disease duration prior to surgical cure [3].

Genetic testing is indicated for suspected familial hyperaldosteronism (FH), although its frequency in clinical practice is low [108]. Genetic studies have revealed somatic mutations in genes like KCNJ5, CACNA1D, ATP1A1, ATP2B3, and CTNNB1 as common drivers of aldosterone overproduction in unilateral APAs [76,82,143]. KCNJ5 mutations, for example, have been reported to be more frequent in APAs from Asian PA patients (60–70%) compared to Western patients (30–40%) [70], though a Malaysian study found a lower prevalence (49.4%) aligning more with Western data [144]. Other mutations such as CACNA1D and ATP1A1 have been implicated in smaller, less cortisol-responsive tumors, often without sex predominance, and are linked to distinct histopathological features, including compact growth and aggressive architecture in some cases [145]. Genetic predisposition to PA susceptibility has been demonstrated in cross-ancestry cohorts, significantly contributing to the genetic background of hypertension [146]. Familial hyperaldosteronism (FH), although uncommon, represents a hereditary origin of PA, with four forms reported (FH types I–IV) [147]. FH-I (glucocorticoid-remediable aldosteronism) involves a chimeric gene between CYP11B1 and CYP11B2, while FH-II is linked to CLCN2 germline mutations; FH-III involves KCNJ5 germline mutations, and FH-IV has been associated with CACNA1H [9,22,148]. Despite the availability of targeted genetic testing for inherited forms (FH-I, FH-III), real-world data suggest that overall screening and testing remain rare in clinical settings, even for patients meeting guideline-based criteria—highlighting a significant gap between recommendations and implementation [3,16,147].

### 3.4. Emerging Associations and Diagnostic Challenges

The increasing prevalence of mild autonomous cortisol secretion (MACS) among PA patients is gaining recognition, with a reported prevalence of 21.9% [149]. MACS in PA is associated with older age, higher plasma aldosterone concentration, lower plasma renin activity, lower eGFR, larger adrenal tumor size, and a higher risk of chronic kidney disease, diabetes mellitus, and cardiovascular diseases [149]. Concurrent MACS further increases the risk of renal complications in PA patients [128]. In addition, specific mutations found in cortisol-producing adenomas (PRKACA, GNAS, CTNNB1) have been investigated in unilateral PA patients with concurrent ACS (uPA/ACS) [150].

## 4. Hyperaldosteronism and Psychiatric Disorders

The possible association between hyperaldosteronism and psychiatric and mental diseases in humans remains frequently overlooked and neglected, despite compelling evidence of significant comorbidity and direct pathophysiological links [151,152]. Lending credence to the multidimensionality of PA, deleterious mineralocorticoid receptor (MR) activation also occurs in the central nervous system (CNS), leading to systemic inflammation, oxidative stress, and fibrosis [153].

Patients with PA frequently present with symptoms of depression and anxiety, with reported prevalence rates as high as 30% and 70%, respectively [151]. Comparative studies show a significantly higher frequency of generalized anxiety disorder in PA patients (30.4%) compared to those with essential hypertension (8.7%) or normotensive controls (0%) [151]. Cross-sectional data indicate that 50% of untreated PA patients exhibit depressive symptoms and anxiety, with these rates remaining substantial even after specific treatment [151]. More recent analyses confirm that a considerable proportion of PA patients meet criteria for depressive symptomatology, with 56% of men and 61% of women affected [135,154]. Furthermore, acute psychiatric manifestations, such as a psychotic episode, can be the inaugural presentation of PA, indicating that clinicians should consider organic causes in such cases [155]. Sleep disturbances, which often accompany depressive and anxiety symptoms, are also pathologically elevated in PA patients, particularly in females [154].

Mechanistically, a direct role of aldosterone in the brain is presumed, where mineralocorticoid receptors (MRs) are expressed in key central nervous system structures, including the hippocampus, amygdala, and prefrontal cortex, as well as brain vessels [152]. While aldosterone concentration in the brain is lower than in plasma, it is proportional to serum levels and appears to be synthesized locally within the CNS [152]. Critical to this action is the enzyme 11β-hydroxysteroid dehydrogenase type 2 (11βHSD2), which inactivates cortisol, thereby enabling aldosterone to selectively bind to MRs in specific brain regions like the nucleus of the solitary tract (NTS) [135,152,154]. The NTS plays a vital role in autonomic, sleep, and affect regulation, with projections influencing mood and cognition [152,154]. Chronically elevated aldosterone levels have been linked to oxidative stress and inflammation in brain tissue, mediated via endothelial cell MRs, and may contribute to glycocalyx damage, leading to cerebrovascular alterations and cognitive decline, independently of blood pressure [152]. In depression, elevated aldosterone levels have even been associated with structural brain changes, including enlarged ventricles, which correlate with poorer treatment outcomes [152].

Gender differences are notably prominent in the psychiatric presentation of PA. Women with PA tend to experience worse sleep quality and higher rates of depressive symptoms [154]. Studies suggest that higher aldosterone concentrations are significantly associated with depressive symptoms in women, a relationship less pronounced in men [135]. Interestingly, females with PA demonstrate similar sleep-EEG patterns to those with major depression, unlike males [154]. Furthermore, BMI is an independent risk factor for depressive symptoms in PA patients of both genders [135], suggesting a complex interplay between hormonal, metabolic, and psychological factors. Specific treatments for PA, such as adrenalectomy or administration of mineralocorticoid receptor antagonists (MRAs), usually lead to significant improvement in sleep disturbance, depression, and anxiety symptoms [154]. Given the substantial prevalence of PA and its profound, yet often unrecognized, psychiatric sequelae, physicians across specialties must be vigilant in screening for and addressing these comorbidities, as early diagnosis and appropriate treatment offer the potential for improved mental health outcomes in this vulnerable patient population [151,154].

## 5. Conclusions

PA is more common than once believed [6], with prevalence conservatively estimated at 30% [6], in appropriately screened subjects. It remains frequently underdiagnosed, with major cardiovascular and renal consequences. Moreover, PA is best considered as a broad continuum of renin-independent aldosterone production [9], rather than a binary disease [9]. The expanding understanding of PA’s diverse clinical phenotypes, genetic mechanisms, and associations with metabolic and other systemic disorders underscores the need for increased awareness among physicians. The caveat is that interpretation of the current evidence requires caution. Most available studies are heterogeneous in design, populations, and diagnostic criteria, and many are derived from tertiary referral centers, which may overestimate prevalence compared with community-based cohorts. Differences in laboratory assays, confirmatory protocols, and even regional practices make comparison between studies difficult. Publication bias may also have influenced the apparent magnitude of associations. The finding that subjects with untreated PA patients have an almost 3-fold higher cardiovascular risk profile than subjects with essential hypertension, matched for blood pressure levels [6], reinforces the need for improved detection [6].

Overall, implementing rigorous and standardized screening protocols, guided by updated guidelines and considering a broader range of risk factors beyond classical hypokalemia, is crucial for early detection and personalized management. Future research should focus on validating optimal screening strategies, elucidating the complex pathophysiological links with associated comorbidities, and improving the accessibility and accuracy of diagnostic tools to mitigate the substantial burden of untreated PA.

## Figures and Tables

**Table 1:** Screening and diagnosis of primary hyperaldosteronism.

Step	Endocrine Society [16]	Korean Endocrine Society [15]	SFE/SFHTA/AFCE Consensus [17]
Who to Screen	Resistant HTN, hypokalemia, adrenal incidentaloma, early-onset HTN, OSA, relevant family history.	Same indications; emphasizes low threshold in primary care due to underdiagnosis.	Targeted screening of classic high-risk groups.
Initial Test	ARR (PAC with PRA or PRC); standardized sampling needed.	ARR (PAC/PRA or PAC/PRC); stresses assay variability.	ARR under standardized conditions.
ARR Cutoffs	No universal cutoff; use assay- and population-specific thresholds.	Same; highlights LC-MS/MS impact on values/cutoffs.	Local assay considerations; adjust for medications.
Confirmatory Testing	Recommended after positive ARR (SIT, FST, OSLT, CCT).	Recommended but often underused; notes variability in test interpretation.	Recommended using standard protocols.
Preferred Tests	SIT, FST, OSLT, CCT.	SIT, FST, CCT; LC-MS/MS aldosterone may improve accuracy.	SIT, FST, CCT acceptable.
Subtyping/AVS	AVS is gold standard before surgery.	AVS strongly recommended; technical enhancements noted.	AVS preferred; imaging alone insufficient.
Role of Imaging/Biomarkers	CT/MRI for anatomy; PET/steroid profiling emerging but not replacements for AVS.	Highlights functional PET and steroid profiling as helpful adjuncts.	Imaging limited; emerging tools not routine.
Medication Handling	Adjust or interpret around interfering antihypertensives.	Medication interference common; adjust when feasible.	Medication standardization improves ARR accuracy.

For the explanation of abbreviations please see the list at the end of the article.

## Data Availability

No new data were created for this work.

## Abbreviations

The following abbreviations are used in this manuscript:

11βHSD2	11β-hydroxysteroid dehydrogenase type 2
APA	aldosterone-producing adenoma
ARR	aldosterone-to-renin ratio
AVS	adrenal venous sampling
baPWV	brachial-ankle pulse wave velocity
BMI	body mass index
CAD	coronary artery disease
CCT	captopril challenge test
CHF	congestive heart failure
CKD	chronic kidney disease
CLIA	chemiluminescence immunoassay
CNS	central nervous system
CT	Computed Tomography
CYP11B2	aldosterone synthase
CXCR4	C-X-C motif chemokine receptor 4
DM	diabetes mellitus
EEG	electroencephalogram
eGFR	estimated glomerular filtration rate
EHTN	essential hypertension
ECV	extracellular volume
FH	familial hyperaldosteronism
FST	fludrocortisone suppression test
IHA	idiopathic hyperaldosteronism
LVH	left ventricular hypertrophy
LC-MS/MS	liquid chromatography-tandem mass spectrometry
MACE	Major Adverse Cardiovascular Events
MACS	mild autonomous cortisol secretion
MetS	metabolic syndrome
MR	mineralocorticoid receptor
MRI	Magnetic Resonance Imaging
NTG	normal-tension glaucoma
NTS	nucleus of the solitary tract
OR	odds ratio
OSA	obstructive sleep apnea
OSLT	oral sodium loading test
PA	primary aldosteronism
PAC	plasma aldosterone concentration
PET/CT	Positron Emission Tomography/Computed Tomography
PRC	plasma renin concentration
PRA	plasma renin activity
PTC	papillary thyroid cancer
RAAS	renin–angiotensin–aldosterone system
RAV	right adrenal vein
RR	relative risk
SIT	saline infusion test
UHR	uric acid-to-high-density lipoprotein cholesterol ratio
